# Deep-frying palm olein oil-fried street falafel induces testicular toxicity in rats

**DOI:** 10.1016/j.toxrep.2023.09.006

**Published:** 2023-09-07

**Authors:** Ahmed Mohamed GadAllah, Mohamad Anwer Noaman, Mohamed nafea Azab

**Affiliations:** Forensic Medicine and Clinical Toxicology, Al-Azhar University, Assiut, Egypt

**Keywords:** Deep-frying, Palm olein oil, Falafel, Testes

## Abstract

Falafel is the most common Egyptian street food, and deep-frying palm oil is a commonly used for frying it. The main objective of this research was to evaluate the effect of deep-frying palm olein oil-fried street falafel on testes of Wistar rats. Twenty-one adult male Wistar rats were randomly divided into three equal groups and given treatment as follows: control group received only distilled water, fresh palm olein oil (FPO) group received FPO (1 ml/100 g BW/day) and deep-frying palm olein oil (DPO) group received DPO (1 ml/100 g BW/day) orally for 28 days. Serum level of testosterone, testicular tissue oxidative status, and sperm characteristics were determined. Testicular specimens were processed for histopathological examination. The results revealed that DPO group showed a significant (*p*<0.01) decrease in serum testosterone with significant (*p*<0.01) reduction of testicular glutathione, superoxide dismutase*,* and glutathione peroxidase, whereas testicular malondialdehyde was markedly (*p*<0.001) raised. There were significant decrease in epididymal sperm count (*p*<0.01), sperm progress motility (*p*<0.001), and increase abnormal sperm rate (*p*<0.001) in DPO group. Testicular histology in DPO group showed structural abnormalities which are compatible with lipid peroxidation and antioxidant deficiency. In Conclusion, deep-frying palm olein oil that used for the frying falafel induces testicular abnormalities in rats.

## Highlights

• Repeatedly heated palm olein oil in deep-frying has detrimental health effects consequences on health.
• Deep-frying palm olein oil induces testicular toxicity.
• Pathogenesis of testicular toxicity includes oxidative stress.
• Knowledge of falafel by that oil is important prepared
• Enhancement and guarantee the safety of street foods prepared by such oils is necessary.

## 1 Introduction

Street food is specifically popular in low and middle-income countries, where it represents an essential part of their economy [1], [2]. However, street food composes a basic part of the consuming behavior in many cities of the high-income countries [3], [4]. According to the FAO definition, street foods are ready-prepared foods by vendors or hawkers especially in the streets and other comparable places [5].

Deep-frying is a worldwide method of food preparation. Frying is a procedure of immersing food in boiling oil with a contact through oil, air, and food at a temperature between 150 ºC and 190 ºC. Frying oil serves as a heat-transfer medium and participates in the texture and taste of fried food [6]. The absorbed oil has a tendency to collect on the exterior of fried food during frying and extend into the interior of food during cooling [7], and consequently absorbed from the gastrointestinal tract to systemic circulation after ingestion. Physicochemical alterations of cooking oil and fried food are harmful. Presence of oxygen, humidity, use of repeatedly heated/recycling oil practice, frying time and temperature, and free radicals contributes to initiate hydrolysis, polymerization and oxidation of oil and lipids and degradation of its physicochemical and antioxidant qualities with generation of oxidative stress and free fatty acids [8], [9], [10], [11], that induces injury at the cellular and molecular levels [12]. Repeated Consumption of deep-frying oil cause deleterious health effects as hypertension [13], increase lipid peroxidation and LDL [14], atherosclerosis [15], risk of cardiovascular diseases [16], impaired liver and kidney functions [17], carcinogenic effects [18].

Palm oil is derived from the mesocarp of tropical plant Elaeis guineensis. It is one of the most common worldwide vegetable oils. Palm oil accounts for more than half of all vegetable fats and oils consumed [19]. The refined, bleached, and deodorized palm olein oil is subdivided from palm oil. It is commonly used for deep-frying and provides a good resistance to oxidation at high temperature throughout the frying; in addition, it contains the natural antioxidants from the vitamin E group, the tocotrienols, as well as, low content of polyunsaturated fatty acids [20].

Falafel (Ta′amiya) is a common Egyptian street food. The basic component is dried fava beans which soaked in water for a period of time then grinded and kneaded with leeks, parsley, onion, garlic and appropriate amount of salt followed by deep-frying in refined palm olein oil in the form of tablets. Some introduce sesame seeds and broken coriander seeds to make it more tasty. A little amount of baking soda is commonly added to falafel dough to increase expansion of tablets. Consumption of deep-fried falafel made in roadside stalls, roadside cafeterias and car parks, and restaurants is extremely common. In Egypt, socioeconomic status of people influences their food consumption pattern, whereas people from low and middle-income groups depending mainly on a morning meal of deep-fried falafel.

However, the current research focus on the effects of repeated ingestion of deep-frying refined palm olein oil-fried street falafel on the testes of rats, to our knowledge, no previous studies in literature discuss the effects of repeatedly heated administration of vegetable oils on reproductive system in human or animals. Therefore, the importance of study′s novelty extends beyond national to international health issue.

## 2 Aims of the current work

The present study aimed to investigate the effect of deep-frying refined palm olein oil-fried street falafel on the testes of rats.

## 3 Materials and methods

### 3.1 Chemicals

Chemicals were purchased from sigmaaldrich.com, products of Merck, Darmstadt, Germany and Sigma (St Louis, MO, USA).

### 3.2 Experimental animals

The study was conducted on 21 mature male Wistar rats weighing between 165 and 195 g. The rats were purchased from animal house of Assiut University, Egypt. The experimental protocol was approved by Faculty of Medicine Ethics Committee of Al-Azhar University, Egypt, and followed the National institutes of Health Guide for the Care and Use of Laboratory Animals. Seven animals were housed in metabolic cages which were kept at a temperature of 22 ± 2 ºC, with relative humidity of 50 ± 5 % and a 12-h/12-h light/dark cycle in accordance with established laboratory animals. They had unrestricted access to fresh water and rat chow. Prior to the start of the trial, the animals were acclimatized for 7 days.

### 3.3 Preparation of deep-frying refined palm olein oil (DPO)

Fresh refined palm olein oil (FPO) was purchased from a local market in Assiut, Egypt. DPO was prepared using a modified method described by Chao et al. [21]. In a wok made of stainless steel, 4 kg of FPO was poured and heated at 180 ºC. About 5 kg of falafel dough tablets were fried in the oil per day. The frying process took 6 h per day and was repeated four times. No additional fresh oil was added after the daily frying procedures to make up for the loss brought on by the fried falafel's absorption.

### 3.4 Experimental design

Following a period of acclimatization, the rats were equally divided into three groups with seven rats in each group. The following treatment plan was used:

Control group: was given distilled water (1 ml/100 g BW/day) orally by gastric tube for 28 days.

FPO group: was given FPO (1 ml/100 g BW/day) orally by gastric tube for 28 days.

DPO group: was given DPO (1 ml/100 g BW/day) orally by gastric tube for 28 days.

Rats were weighed and recorded at the start of the experiment and every week until the final body weight. Animals were fasted overnight for 12 h at the study's end (4 weeks) and then euthanized by giving sodium pentobarbital (200 mg/kg IP) [22], [23] anesthesia.

### 3.5 Collection and preparation of tissue

Immediately, blood was extracted from the heart into plain test tubes and allowed to clot. The serum was separated from the clotted blood samples using a centrifugation at 3000 g for 15 min, and the serum were then maintained at – 20 ºC until the serum testosterone was measured. The left testis of each rat was immediately removed, immediately split into two pieces, and weighed to the nearest milligram using an electronic Shimadzu balance (BL-220 H; Kyoto, Japan). One portion was used for biochemical assaying (lipid peroxidation and antioxidant enzymes), and the other was blocked for histopathological examination. Testicular tissues were perfused with a PBS (phosphate buffered saline) solution, pH 7.4, containing 0.16 mg/ml heparin to remove any red blood cells and clots, followed by homogenization (10 % w/v) in ice-cold potassium phosphate buffer, pH 7.4, containing 1 mM EDTA, in a Potter-Elvehjem type homogenizer. The homogenate was centrifuged at 4000 g for 15 min at 4 ºC, and supernatants were removed for biochemical analysis.

### 3.6 Biochemical analysis

Lipid peroxidation (Malondialdehyde, an end-product of the lipid peroxidation), was assayed by the method of Ohkawa et al.[24], based on thiobarbituric acid (TBA) reacts with malondialdehyde (MDA) in acidic medium at temperature of 95 ºC for 30 min to form thiobarbituric acid reactive product the absorbance of the resultant pink product can be measured at 534 nm. Reduced glutathione (GSH) level was assayed by the method of Beutler et al.[25], based on the reduction of 5,5′ dithiobis (2-nitrobenzoic acid) (DTNB) with glutathione (GSH) to produce a yellow compound. The reduced chromogen directly proportional to GSH concentration and its absorbance can be measured at 405 nm. Superoxide dismutase (SOD) activity was measured by the method of Nishikimi.[26], relies on the ability of the enzyme to inhibit the phenazine methosulphate-mediated reduction of nitroblue tetrazolium dye. Glutathione peroxidase (GSH-Px) activity was measured by the method of Chiu et al.[27], based on the reaction between glutathione remaining after the action of GSH-Px and 5,5′ dithiobis (2-nitrobenzoic acid) to form a complex that absorbs maximally at 412 nm. The serum level of testosterone was measured using enzyme-linked immunosorbant assay (ELISA) kits according to the manufacturer's instructions.

### 3.7 Epididymal sperm count, motility and morphology

Linder et al. method [28] was used to calculate the epididymal sperm count. The epididymis was therefore minced with anatomical scissors in 5 ml of physiological saline and incubated at 32 ºC for 2 min to obtain epididymal spermatozoa. An aliquot of this solution was added to Malassez cells and motile sperm were counted under a 400× microscopic magnification. The total amount of sperm was then tallied after the non-motile sperm counts. The percentage of motile sperm among the total number of sperm counted was used to depict sperm motility.

The percentage of morphologically abnormal spermatozoa was calculated as described by Terpsidis et al. [29] with a slight modification (a drop of the sperm suspension was placed onto a fresh glass plate for each sample and was allowed to dry in air).

### 3.8 Histopathological examination

Fresh testicular tissues were preserved in Bouin's solution for the histological analysis under a light microscope. The specimens were cleaned after being fixed for two days, dehydrated using graded series of ethanol, and then embedded in paraffin wax. Using a rotary microtome, blocks were created and sectioned at a thickness of 4 mm. In distilled water, sections were rehydrated and stained with hematoxylin-eosin and examined under a light microscope [30].

### 3.9 Statistical analysis

All results are expressed as mean ± SD. Data were analyzed by one-way analysis of variance (ANOVA) followed by Turkey's post hoc test. Graph Pad Software Inc., USA, version 7 was used to conduct the statistical analysis. Statistical significance was decided at *p*˂0.05.

## 4 Results

### 4.1 Animal observation, body weight, and weight of the testes

**Fig. 1:**
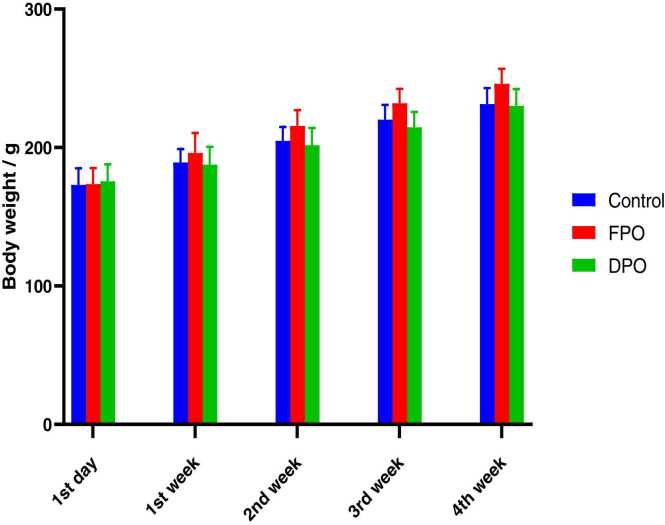
Effect of FPO and DPO versus control group on the body weight of rats. Bars are means ± SD; n = 7 for each group. It shows no statically significant difference between experimental groups(*p*˂0.05).

No deaths and abnormal manifestations were observed after 4 weeks of the experimental period. As shown in Fig. 1 in 1st, 2nd,3rd and 4th week of the experiment no significant changes were detected in the body weight of FPO (173.6 ± 11.61, 196 ± 14.50, 215.6 ± 11.37, 232 ± 10.36 and 246 ± 10.83 respectively) and DPO (175.6 ± 12.28, 187.6 ± 12.91, 201.6 ± 12.54, 214.6 ± 11.10 and 230 ± 12.24 respectively) versus control group (173 ± 12.04, 189.2 ± 9.67, 204.8 ± 10.03, 220 ± 10.79 and 231.4 ± 11.61 respectively). There is slight increase in body weight of FPO group but not significant.

**Fig. 2:**
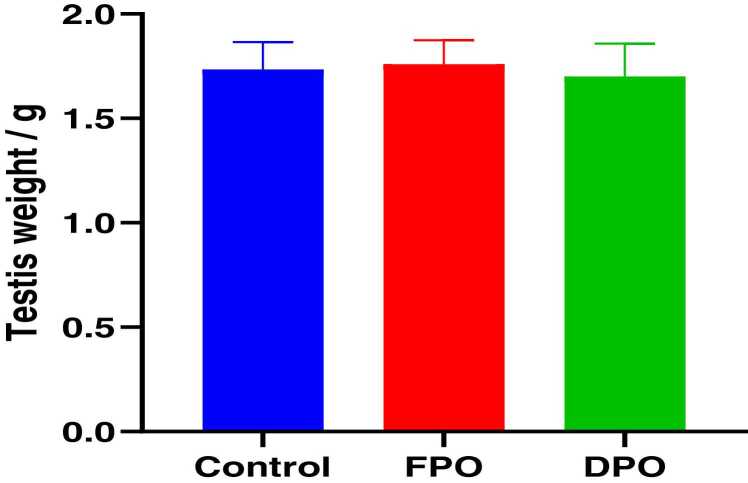
Effect of FPO and DPO versus control group on the testicular weight of rats. Bars are means ± SD; n = 7 for each group. It shows no statically significant difference between experimental groups (*p*˂0.05).

Fig. 2 Shows no significant changes were detected in testicular weight of FPO and DPO versus control group (1.76 ± 0.11 and 1.7 ± 0.15 respectively vs. 1.734 ± 0.13).

### 4.2 Biochemical parameters

#### 4.2.1 Lipid peroxidation and antioxidant enzymes levels

**Fig. 3:**
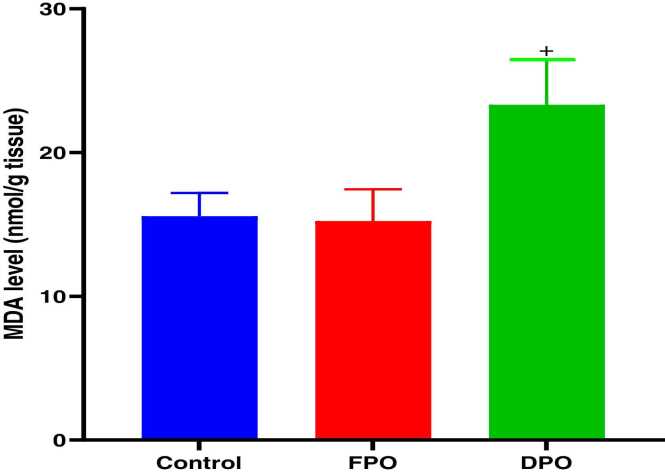
Effect of DPO and FPO against control group on the level of testicular lipid peroxidation in rats. Bars are means ± SD; n = 7 for each group. Data were analyzed by one-way ANOVA followed by Turkey's post hoc test. Significant difference versus control group (^*+*^*p*<0.001).

**Fig. 4:**
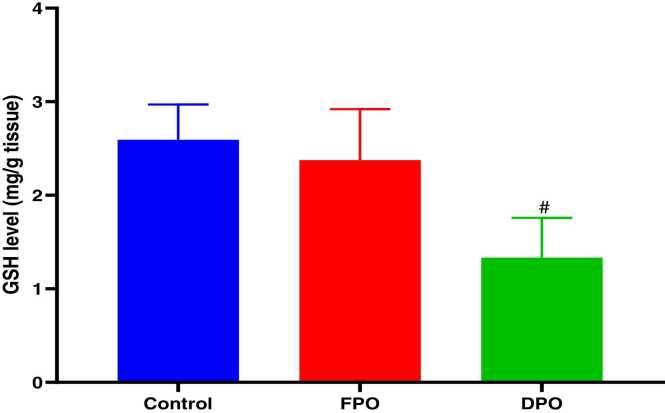
Effect of DPO and FPO on the level of testicular glutathione in rats versus control group. Bars are means ± SD; n = 7 for each group. Data were analyzed by one-way ANOVA followed by Turkey's post hoc test. Significant difference against control group (^*#*^*p*<0.01).

**Fig. 5:**
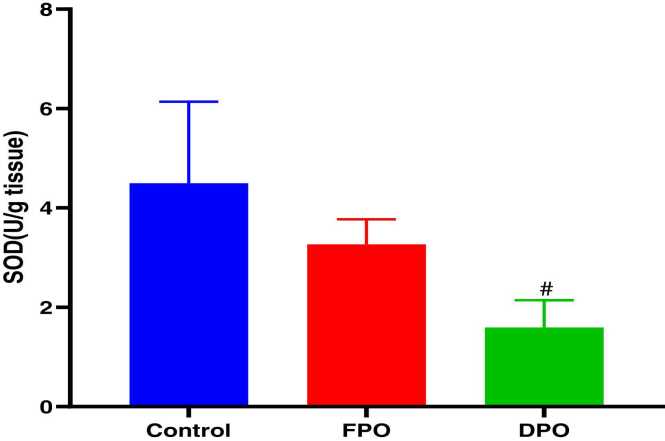
Effect of DPO and FPO on the activity of testicular superoxide dismutase in rats compared to control group. Bars are means ± SD; n = 7 for each group. Data were analyzed by one-way ANOVA followed by Turkey's post hoc test. Significant difference against control group (^*#*^*p*<0.01).

**Fig. 6:**
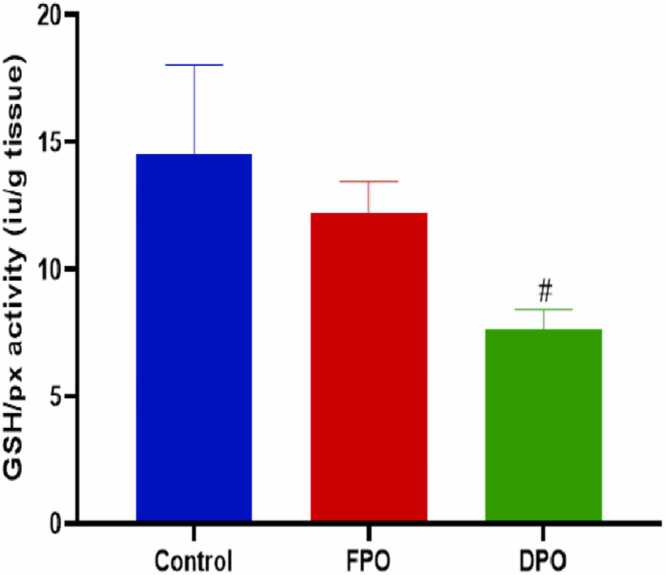
Effect of DPO and FPO on the activity of testicular glutathione peroxidase in rats against control. Bars are means ± SD; n = 7 for each group. Data were analyzed by one-way ANOVA followed by Turkey's post hoc test. Significant difference versus control group (^*#*^*p*<0.01).

Administration of DPO for 28 days exerted a significant increase of testicular MDA level when compared to control group (23.33 ± 3.1 vs. 15.58 ± 1.61; *p*<0.001) whereas administration of FPO failed to exert any significant effect versus control group (15.23 ± 2.21 vs. 15.58 ± 1.61) (Fig. 3). DPO significantly depleted testicular GSH level versus control group (1.33 ± 0.42 vs. 2.59 ± 0.37; *p*<0.01) while no significant effect of FPO on testicular level of GSH when compared to control (2.38 ± 0.54 vs 2.59 ± 0.37) (Fig. 4). The rats administered DPO had significant reduction in activities of SOD and GSH-Px in comparison with control group (1.59 ± 0.54 and 7.66 ± 0.74 vs. 4.49 ± 1.64 and 14.5 ± 3.54 respectively; *p*<0.01) (Fig. 5) (Fig. 6), but no significant effect for FPO on the activities of SOD and GSH-Px against control (3.27 ± 0.50 and 4.49 ± 1.64 and 12.22 ± 1.25 vs. 14.5 ± 3.54 respectively).

#### 4.2.2 Serum level of testosterone

**Fig. 7:**
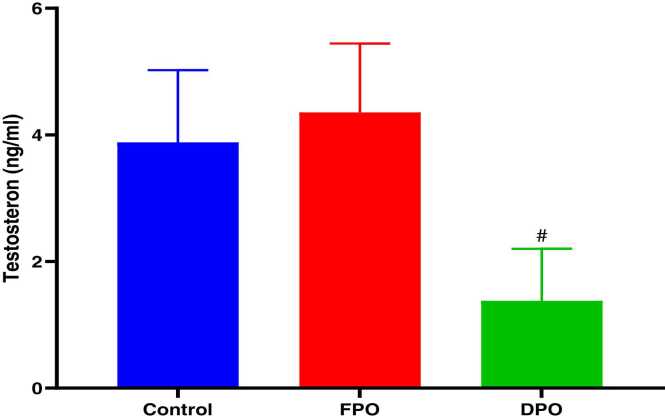
Effect of DPO and FPO on the serum level of testosterone in rats in comparison with control group. Bars are means ± SD; n = 7 for each group. Data were analyzed by one-way ANOVA followed by Turkey's post hoc test. Significant difference versus control group (^*#*^*p*<0.01).

Fig. 7 shows that administration of DPO statistically decreased serum level of testosterone as compared with control (1.38 ± 0.82 vs. 3.88 ± 1.14; *p<*0.01) without any significant effect for FPO in comparison with control group (4.35 ± 1.08 vs. 3.88 ± 1.14) after 28 days of administration.

### 4.3 Sperm parameters

**Fig. 8:**
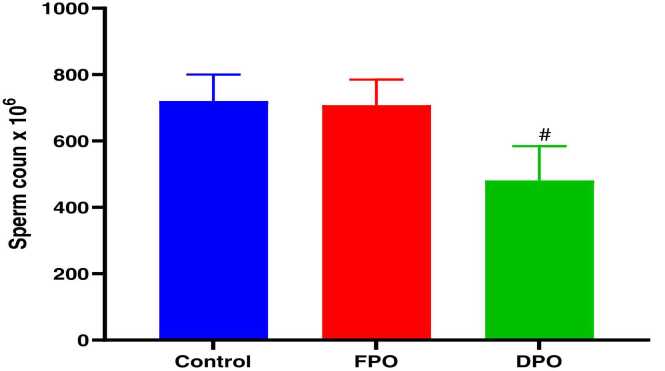
Effect of DPO and FPO on epididymal sperm count in rats compared to control group. Bars are means ± SD; n = 7 for each group. Data were analyzed by one-way ANOVA followed by Turkey's post hoc test. Significant difference against control group (^*#*^*p*<0.01).

**Fig. 9:**
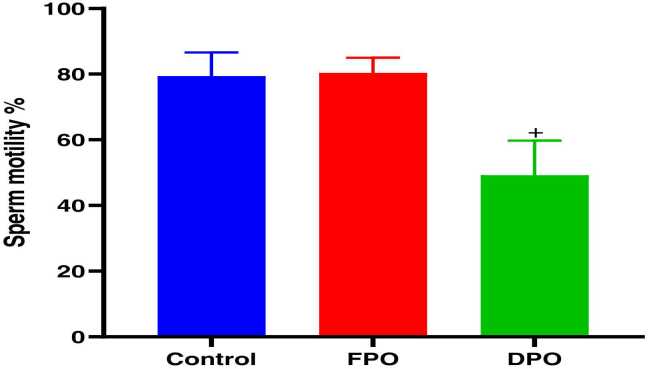
Effect of DPO and FPO on sperm motility in rats in comparison with control group. Bars are means ± SD; n = 7 for each group. Data were analyzed by one-way ANOVA followed by Turkey's post hoc test. Significant difference versus control group (^*+*^*p*<0.001).

**Fig. 10:**
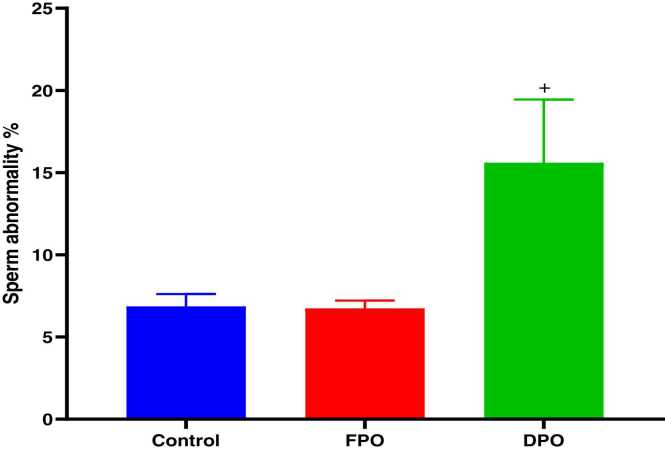
Effect of DPO and FPO on sperm abnormality % rate in rats against control group. Bars are means ± SD; n = 7 for each group. Data were analyzed by one-way ANOVA followed by Turkey's post hoc test. Significant difference versus control group (^*+*^*p*<0.001).

Epididymal sperm count (Fig. 8), sperm motility (Fig. 9), and abnormal sperm rate (Fig. 10) are shown for DPO, FPO and distilled water (control) administration for 28 days. In comparison with control group there were significant decreases in epididymal sperm count (481.3 ± 103.2710^6^ vs. 720.6 ± 79.69 10^6^; *p*<0.01), sperm progress motility (49.2 ± 10.57 % vs. 79.4 ± 7.19 %; *p*˂0.001), and increase abnormal sperm rate (15.6 ± 3.84 % vs, 6.86 ± 0.75 %; *p*˂0.001) with chronic administration of DPO whereas administration of FPO failed to exert any significant effect of sperm count, motility and abnormal rate when compared with control (708 ± 76.9410^6^ 80.4 ± 4.56 % and 6.74 ± 0.47 % vs. 720.6 ± 79.69 10^6^, 79.4 ± 7.19 % and 6.86 ± 0.75 % respectively).

**Fig. 11:**
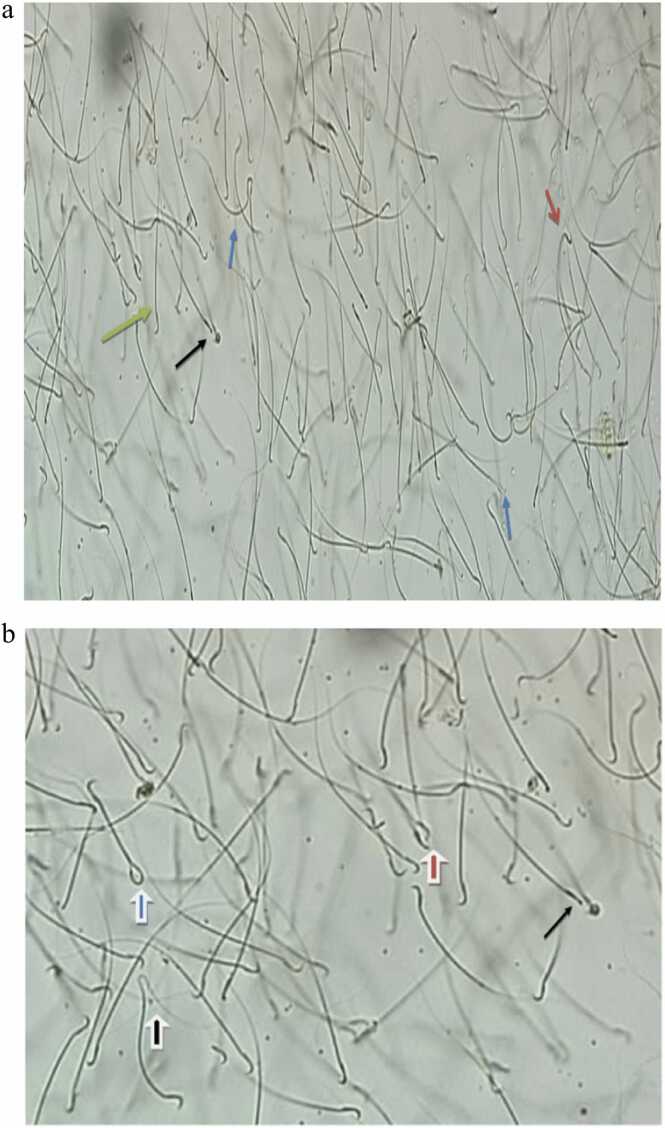
a: Photomicrograph of DPO group shows normal sperm (green arrow), bent neck (red arrow), curved body (blue arrow) and straight head (black arrow) (x 100). b: Photomicrograph of DPO group shows straight head (black arrow) double head (red head arrow), bent tail (black head arrow) and coiled tail (blue head arrow) (x 100).

Fig. 11 shows a normal and some detected abnormal sperm morphology as a result of the chronic administration of DPO.

### 4.4 Histopathological examination

**Fig. 12:**
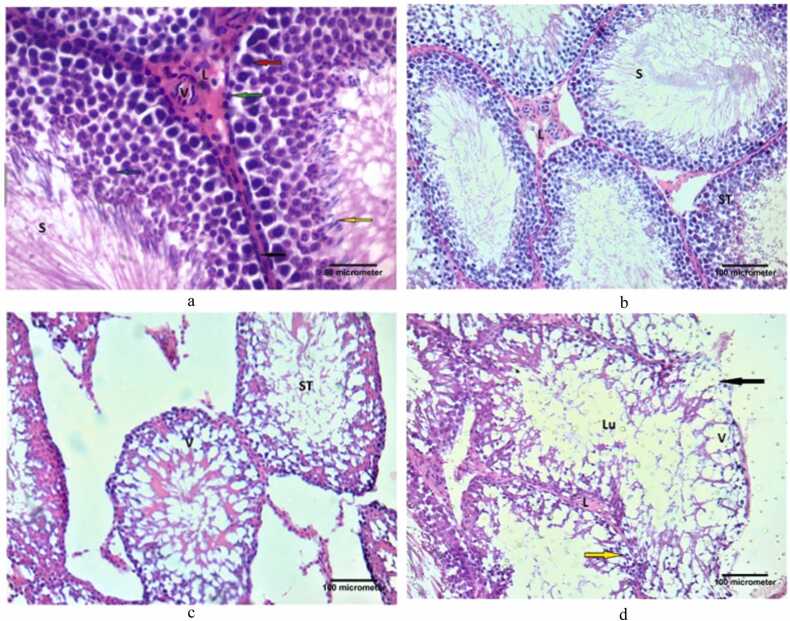
a: Photomicrograph of testicular section of the control group, showing seminiferous tubules surround by regular basement membrane and myoid cell (black arrow). These tubules lined with many layers of spermatogenic cells. The spermatogenic cells are; spermatogonia with dark small nuclei (green arrow), primary spermatocyte, with large vesicular nucleus (red arrow) and spermatid (blue arrow). The head of sperm (yellow arrow) can be seen. The sperm (s) can be seen filled the lumen of the tubule. Notice, the interstitial cell of Leydig (L) which present in groups with acidophilic cytoplasm. Also, there is blood vessel (V) within the interstitial tissue (H&E X 400, scale bar = 50 µm). b: Photomicrograph of testicular section of FPO group showing that the histological structure of the testis is nearly as the control group (H&E X 400, scale bar = 100 µm). c: Photomicrograph of testicular section of DPO group, showing seminiferous tubules appear widely separated and irregular in shape. Multiple vacuolated areas (V) within the seminiferous tubules can be seen (H&E X 400, scale bar = 100 µm).d: Photomicrograph of testicular section of DPO group, showing that the spermatogenic cells are disorganized and apparently decreased in number. These cells have vacuolated cytoplasm and pyknotic; small darkly stained nuclei (yellow arrow). Wide space and vacuolated areas (V) can be seen within the tubules. The lumen of the tubules is wide (Lu) with no sperm can be seen within it. The basement membrane of the seminiferous tubules appears interrupted and discontinuous (black arrow) the interstitial cells of Leydig (L) are apparently decreased in numbers. (H&E X 200, scale bar = 100 µm).

Sections from testes of rats of control, FPO and DPO groups are shown in Fig. 12. The seminiferous tubules in the testes of control rat were lined with multiple layers of spermatogenic cells, which were organized from the basement membrane toward the lumen of tubules. Spermatogonia with dark small nuclei, primary spermatocytes with large vesicular nuclei and spermatids made up the spermatogenic cells. Sperms seen within the lumen of tubules. The interstitial spaces were filled with blood capillaries and clusters of interstitial Leydig cells (Fig. 12a). The FPO group displayed a comparable pattern (Fig. 12b). In the DPO-administered group, the seminiferous tubules appear widely separated and irregular in shape (Fig. 12c), the spermatogenic cells are disorganized and apparently decreased in number. These cells have vacuolated cytoplasm and pyknotic. The lumen of tubules is wide with no sperms can be seen within it. The basement membrane appears interrupted and discontinuous. The interstitial cells of Leydig are apparently decreased in number (Fig. 12d).

## 5 Discussion

To reduce food preparation costs, cooking oil tend to be used repeatedly. Accordingly, the usage of repeatedly heated refined palm olein oil in deep-frying falafel which is a traditional Egyptian street food is common without considering the detrimental consequences on health. However, to our knowledge, there isn't any literature report on the impact of DPO on rat testes. Therefore, the current study is novel. Our research is predicated on the hypothesis that using DPO generates free radicals, which lead to oxidative stress and tissue damage. Testicular tissue is especially vulnerable to oxidative stress due to rapid rate of cell division and high mitochondrial oxygen consumption. Additionally, there is a fierce battle among cells for oxygen since the testicular artery is weak and the oxygen pressure is low [31]. Oxidative stress represents an imbalance between reactive oxygen species (ROS) generation and endogenous antioxidant systems that are used to prevent oxidative damage. Rats consumed DPO seemed to be more susceptible to oxidative stress.

In the current investigation, rats receiving DPO had a significant high level of MDA in their testes. MDA is used as a marker to determine the rate of oxidative damage to lipids and an indicator of cell injury. The elevation of lipid peroxidation may indicate that DPO increases oil oxidation. Leong et al. [32] reported that increased plasma lipid peroxidation may be linked to increased peroxide value of DPO. Even though vitamin E, a powerful lipophilic antioxidant, is abundant in palm oil, its effect may be greatly affected by repeated heating of the oil. Oxidative damage to lipid structures can eventually result in malfunction and disorganization, as well as damage to membranes, enzymes, and proteins [33]. Lipid peroxidation subsequently disrupts ions permeability and fluidity, inactivates membrane-bound receptors or enzymes, and compromises membrane activities, all of which eventually result in membrane rupture [34]. The damage caused by lipid peroxidation, according to Peltola et al. [35], is the primary cause of testicular dysfunction.

A complex network of antioxidant defense mechanisms, including enzymatic and non-enzymatic components like SOD, GSH-Px, and GSH, have been established in the testes. The data from this investigation demonstrated a considerable decline in SOD and GSH-Px activities together with GSH depletion. SOD, GSH-Px, and catalase are regarded as first-line enzymatic antioxidants [36], although catalase has a limited role in the testes [37]. These antioxidant enzymes' primary function is to quickly convert superoxide radicals into hydrogen peroxide (H_2_O_2_) in the presence of SOD. Superoxide radical is the most easily generated free radical due to increased mitochondrial oxygen consumption during spermatogenesis in the testis [38] or phagocytosis of germ cell debris in the testis by testicular somatic cells, producing superoxide radical by-products [39]. Furthermore, according to Fenton reaction, H_2_O_2_ produced in the presence of Fe^2+^ can be changed into hydroxyl radical (*OH), which is another toxic free radical [36]. As a result, the H_2_O_2_ produced is quickly removed from the cell to protect DNA, lipids, and proteins from oxidative damage. GSH-Px is able to eliminate H_2_O_2_ by using reduced glutathione (GSH) as the electron donor producing H_2_O [33].

Glutathione, a major cellular antioxidant, is essential for preventing cell against oxidative damage. While reducing H_2_O_2_ and other peroxides, GSH directly interacts with ROS and serves as a cofactor for GSH-Px [31], [40]. According to a study, 5 mM of GSH increases sperm motility after freezing and has protective effects on sperm against freezing. Additionally, this substance enhances enzymatic activity in sheep semen [41].

In the current study, the characteristics of sperm were examined to assess the effects of DPO on spermatogenesis in rat testes. The findings showed that in rats receiving DPO, sperm concentration and motility drastically decreased, along with a considerable rise in sperm abnormalities. Due to the sperm's small volume of cytoplasm, concentration of ROS-suppressing antioxidants, and high levels of unsaturated fatty acids in its structure, researchers think that sperm are more vulnerable to oxidative stress than other cells [41]. The production and increase of abnormal sperm, as well as the reduction in sperm count, and transformation and fragmentation of sperm DNA, are all considerably influenced by the oxidative stress caused by free radicals [42]. Additionally, generation of ROS by leukocytes or spermatozoid impair the sperm function in infertile men's [31].

Testosterone synthesized in Leydig cells is a key component in the growth of the male reproductive system and spermatogenesis. In our study, the serum levels of testosterone were significantly decreased in DPO-administered animals. Free radicals and ROS can affect testicular steroidogenesis, and there is a link between the formation of free radicals and gonadal steroidogenesis [43]. As a result, the fall in testosterone levels in the rats that received DPO may be attributed directly to oxidative stress.

Histological study of testes of DPO-administered rats revealed variable degrees of degeneration in the seminiferous tubules. They showed widely separated and irregular with interruption of basement membrane, disorganization, and vacuolization of spermatogenic cells. The lumen of tubules is wide with no sperms can be seen within it and interstitial cells of Leydig are apparently decreased in number. These findings are probably due to oxidative stress and low testosterone level. In order for sperm to grow normally, testosterone is required. According to Asiyah et al. [44], testosterone plays a significant role in spermatogenesis by serving as the primary hormone for spermatogonia conversion and spermatid production. Therefore, a decline in testosterone levels will result in male sterility.

## 6 Conclusion

The results of this study provide solid evidence that DPO causes testicular injury (decrease serum testosterone level, decrease sperm count and motility, increase abnormal sperm morphologies, and histological abnormalities) by increasing oxidative stress, which may be caused by an excess of ROS generation and a decline in antioxidant capacity.

Further studies are needed to evaluates the quality of deep-frying oils from street-food vendors in Egypt. Strict observation of food street vendors by health government authorities is critical. Health awareness of public regarding the detrimental effects of the use of repeatedly heated cooking oils. Studies are also needed to show whether DNA abnormalities were incorporated in pathogenesis of testicular toxicity induced by DPO. Finally, studies are needed to show safe dose and duration of exposure to heated oil and comparison between different cooking oils and different temperature.

## 7 Limitations

Although, the study achieved its goal there is some limitations. Firstly, in spite of the sample sizes were sufficient for the exploratory nature of the study, the small sample size limits generalization [45]. Secondly, the study discusses the harmful effect of (DPO) but gives no solution for the problem. Thirdly, the study observes the effect of heated oil but people are not exposed directly to heated oil but to the food which is prepared by such oil. Lastly, the study doesn’t show the safe dose (dose response relationship) and safe period of exposure (duration response relationship).

## CRediT authorship contribution statement

**Ahmed Mohamed GadAllah** and **Mohamad Anwer Noaman** were principal investigators of the study and drafted the manuscript. **Ahmed Mohamed GadAllah** was advisors of the study. **Mohamad Anwer Noaman** performed the statistical analysis. **Mohamad Anwer Noaman** and **Mohamed nafea Azab** performed the methodology. All authors contributed to the design and data analysis and assisted in the preparation of the final version of the manuscript. All authors read and approved the final version of the manuscript.

## Declaration of Competing Interest

The authors declare that they have no known competing financial interests or personal relationships that could have appeared to influence the work reported in this paper.

## Data Availability

Data will be made available on request.
